# Genre-Specific Gaming Addiction and Flourishing in Adolescents: Cross-Sectional Survey Study

**DOI:** 10.2196/89319

**Published:** 2026-02-12

**Authors:** Yan Luo, Xiaofei Chen, Yaoyu Liu, Wanjia Hong, Miner Fan, David Thomas, Sean P Mullen

**Affiliations:** 1 Department of Physical Education Shanghai Jiao Tong University Shanghai, Shanghai China; 2 Informatics Programs University of Illinois Urbana-Champaign Urbana, IL United States; 3 Department of Health and Kinesiology University of Illinois Urbana-Champaign Urbana, IL United States; 4 Beckman Institute for Advanced Science and Technology University of Illinois Urbana-Champaign Urbana, IL United States

**Keywords:** digital game, online game, problematic gaming, gaming disorder, youth

## Abstract

**Background:**

Adolescent gaming addiction (GA) has been linked to a range of adverse health outcomes. However, whether the associated health risks differ across game genres remains poorly understood.

**Objective:**

Guided by VanderWeele’s multidimensional flourishing framework, this study aims to examine genre-specific associations between GA and flourishing among adolescents.

**Methods:**

This study used a cross-sectional observational design. A total of 2194 middle school students were recruited via convenience sampling from a private tutoring center in a northwestern city in China. Eligibility criteria were (1) enrollment in participating classes at the tutoring center, (2) provision of both student and parental consent, and (3) presence during questionnaire administration. The mean age of participants was 14.53 (SD 0.76) years; 985 (44.90%) were boys and 1174 (53.51%) were girls. During class time, students completed paper-based questionnaires that assessed their demographics, gaming addiction, and flourishing. Participants listed up to 3 video games played in the past month and rated their addiction to each. Games were classified into 8 genres: action and adventure (AA), sandbox and simulation (SS), multiplayer online battle arena (MOBA), shooting, strategy, casual, sports, and role-playing. Flourishing was assessed using the Human Flourishing Index across 5 domains: happiness and life satisfaction, mental and physical health, meaning and purpose, character and virtue, and close social relationships.

**Results:**

Robust linear regression analyses (α=.05) showed that AA addiction was associated with lower overall flourishing (b=–3.11, 95% CI –4.34 to –1.88) and all 5 subdomains (happiness and life satisfaction: b=–0.46, 95% CI –0.75 to –0.17; mental and physical health: b=–0.61, 95% CI –0.88 to –0.34; meaning and purpose: b=–0.55, 95% CI –0.82 to –0.27; character and virtue: b=–0.74, 95% CI –1.06 to –0.43; and close social relationships: b=–0.62, 95% CI –0.92 to –0.32). MOBA addiction was associated with lower overall flourishing (b=–1.33, 95% CI –2.34 to –0.32), character and virtue (b=–0.34, 95% CI –0.59 to –0.08), and meaning and purpose (b=–0.34, 95% CI –0.56 to –0.11). SS addiction was associated with lower overall flourishing (b=–3.42, 95% CI –5.80 to –1.04), close social relationships (b=–0.86, 95% CI –1.46 to –0.27), and mental and physical health (b=–1.09, 95% CI –1.60 to –0.58).

**Conclusions:**

This study provides novel evidence that the association between GA and adolescent flourishing is genre dependent. In contrast to prior research that conceptualizes health narrowly or unidimensionally, a multidimensional perspective provides a more nuanced understanding of the health risks associated with GA. The findings advance the field by showing that addiction to AA, MOBA, and SS games is associated with greater health risks than addiction to other genres. Accordingly, prevention, education, and policy efforts should prioritize higher-risk genres to promote adolescent health.

## Introduction

### Background

Video gaming is one of the most prevalent leisure activities among adolescents worldwide. For many, gaming provides entertainment, opportunities for social interaction, and cognitive benefits [1]. Yet, for a notable proportion of adolescents, gaming becomes addictive, characterized by preoccupation, impaired control, and persistence despite negative consequences. A meta-analysis estimated the prevalence of gaming disorder, a severe and clinical form of gaming addiction (GA), among adolescents to be 8.8%, based on over 400,000 participants from 155 studies across 33 countries [2]. This rate is considerably higher than that observed in other age groups [3]. Therefore, it is critical to investigate the associations between GA and adolescents’ health to inform interventions and policies that foster healthier gaming practices.

A substantial body of research has documented that GA is associated with a range of negative health outcomes in adolescents [2,4]. Although these studies have significantly advanced our understanding of GA-related harms, a notable limitation is that most have adopted a relatively narrow conceptualization of health, focusing disproportionately on pathological conditions. According to the World Health Organization, health is defined as “a state of complete physical, mental, and social wellbeing” [5], which highlights the need to investigate the relationship between GA and health from a broader perspective. One construct that captures this view is flourishing, which reflects a state in which “all aspects of a person’s life are good” [6]. VanderWeele [6] proposed that flourishing should, at a minimum, encompass 5 domains of human life: (1) happiness and life satisfaction, (2) mental and physical health, (3) meaning and purpose, (4) character and virtue, and (5) close social relationships [6]. Collectively, these domains reflect distinct facets of a well-lived life. Happiness and life satisfaction reflect how good life feels overall. Mental and physical health capture how well individuals are functioning psychologically and biologically. Meaning and purpose speak to whether life feels worthwhile and directed. Character and virtue reflect the capacity for moral self-regulation and for acting well when facing challenges. Close social relationships reflect the quality of one’s relational world. Although these domains are often correlated, they are conceptually distinct. Flourishing entails doing well across all 5 domains, and impairment in any single domain indicates a substantive shortfall in overall well-being. Therefore, examining the association between GA and flourishing is crucial, as it provides a more holistic understanding of how GA may constrain adolescents’ ability to achieve well-rounded lives and may inform prevention and policy efforts to promote adolescents’ health.

### Review of Relevant Scholarship

Previous research suggests that GA is associated with multiple flourishing-related domains. In the domain of happiness and life satisfaction, a large cross-national study of over 14,000 adolescents found that GA was linked to lower life satisfaction [7]. A longitudinal study further showed that GA predicted subsequent declines in adolescents’ life satisfaction over 6 months, whereas the reverse effect was not observed [8]. These findings suggest that GA may function as an antecedent of reduced life satisfaction. Although no studies to date have directly examined the association between GA and happiness among adolescents, GA has been consistently shown to correlate with several factors strongly connected to happiness [9-13], including poorer academic achievement [2], lower self-esteem [2], reduced health-related quality of life [14], more frequent negative life events [15], and worse family financial conditions [16]. In summary, existing evidence suggests that GA is associated with lower life satisfaction and may also be linked to lower levels of happiness among adolescents.

The relationship between GA and mental health outcomes has been the most extensively investigated. Studies using composite indicators of mental health have shown that GA was associated with more frequent psychological complaints [7], elevated psychopathological symptoms [17-19], and a higher likelihood of receiving at least one mental health diagnosis [20]. Research focusing on specific mental health conditions further revealed that GA was associated with increased depression [2,21], anxiety [22-24], general and academic stress [2,25,26], loneliness [27], emotional distress [2,23,28,29], suicidal ideation [19,30], suicide risk [31], emotional and behavioral problems [24], sleep difficulties [4,32], and executive dysfunction [32]. A few studies have examined the association between GA and physical health. Evidence suggests that GA is linked to unhealthy weight status, including both underweight and overweight [33,34], more somatic complaints [17], musculoskeletal pain [4], and dry eye symptoms [4]. Overall, these findings suggest that GA is associated with a broad range of mental and physical health problems, with the literature to date focusing predominantly on mental health outcomes.

Evidence supporting associations between GA and the remaining flourishing domains is limited in the current literature. Existing findings suggest that adolescents with GA reported both a lower presence of meaning in life and a stronger search for meaning in life [35]. Although no adolescent studies have directly examined GA in relation to sense of purpose, research with young adults indicates a negative association between GA and purpose in life [36,37]. Regarding character and virtue, studies have shown that GA is linked to lower empathy [38], lower moral levels [39], reduced academic perseverance [40], and fewer prosocial behaviors [38]. In the relational domain, GA has been associated with lower interpersonal trust [41], conflictual parent-child relationships [42,43], peer alienation [26], less social support [44], and greater reliance on online-only friendships [14]. Overall, the available evidence suggests that GA may have broad adverse associations across these flourishing domains in adolescents.

Beyond research conducted on individual domains of flourishing, no study to date has investigated GA in relation to flourishing as an integrated construct. Existing work on other problematic behaviors has found that lower flourishing was related to greater cannabis use [45], higher media addiction [46], higher smartphone addiction [47], and higher Facebook addiction [48,49]. These findings imply that GA, as a form of problematic behavior, may likewise be associated with reduced flourishing. It is important to note that the flourishing framework used in these studies is derived from Diener and colleagues [50], which captures all but the health dimension of flourishing proposed by VanderWeele [6]. As a result, it remains unclear whether VanderWeele’s broader conceptualization of flourishing is associated with any form of problematic behavior, including GA. A recent study that employed VanderWeele’s flourishing framework examined the relationship between digital game use and flourishing among older adults. No differences were found in overall flourishing or in any of the 5 subdomains between current gamers, former gamers, and nongamers [51]. As this study did not focus on GA and was conducted in an older adult population, its findings provide minimal guidance regarding how GA relates to flourishing among adolescent gamers.

Although prior studies have documented associations between GA and individual elements of flourishing, a critical gap remains regarding how GA relates to overall flourishing and its subdomains among adolescents. As flourishing is a multidimensional construct encompassing various aspects of well-being, investigating these associations is essential for establishing a more comprehensive understanding of how GA may relate to adolescents’ well-being across multiple life domains. A further limitation of the existing literature is that most studies have conceptualized gaming as a single, homogeneous activity. It remains unclear whether GA is uniformly associated with flourishing across different game genres. Emerging evidence suggests that the psychological correlates of gaming may vary substantially by genre. For example, Park and colleagues [52] reported that adolescents who primarily played massively multiplayer online role-playing games exhibited the highest levels of social anxiety, those who preferred first-person shooter games showed the lowest anxiety levels, and strategy-game players reported the highest self-esteem [52]. Other studies have demonstrated that certain genres were associated with increased aggression, reduced empathy, and prosocial behaviors [53-56]. As these factors are important components of flourishing, such findings suggest that the association between GA and flourishing may differ depending on the game genres adolescents play. Understanding these genre-specific associations could provide a more nuanced picture of how GA relates to adolescent health, thereby informing the development of targeted intervention strategies tailored to specific game genres.

### Hypothesis and Aim

To address these gaps, this study aimed to investigate genre-specific associations between GA and flourishing among adolescents, using VanderWeele’s multidimensional flourishing framework. Drawing on prior evidence, we specified a single primary hypothesis: *GA would be negatively associated with overall flourishing and its subdomains for certain game genres, but not uniformly across all genres.* Our secondary hypothesis was that *overall GA across game genres would be negatively associated with overall flourishing and its subdomains.*

## Methods

### Inclusion and Exclusion

This study was reported in accordance with the Journal Article Reporting Standards for Quantitative Research (JARS-Quant) checklist [57]. A total of 2194 middle school students from a private tutoring center located in a northwestern city in China participated in this study. Inclusion criteria were as follows: (1) enrollment in participating classes at the tutoring center; (2) provision of both student and parental consent; and (3) presence during questionnaire administration. No exclusion criteria were applied.

### Participant Characteristics

As shown in Table 1, participants had a mean age of 14.53 (SD 0.76) years. Of the 2194 students, there were 985 (44.90%) boys and 1174 (53.51%) girls. Regarding grade level, 52 (2.37%) were in the seventh grade, 969 (44.17%) were in the eighth grade, and 1092 (49.77%) were in the ninth grade. On average, participants reported a family economic status of 2.23 (SD 0.70) and 18.81 (SD 16.40) hours of social media use per week. In terms of gaming behavior, 610 (27.80%) participants reported not playing any games, 327 (14.90%) reported playing 1 game, 525 (23.93%) reported playing 2 games, and 732 (33.36%) reported playing 3 games. The most frequently played game genre was multiplayer online battle arena (MOBA; n=808, 36.83%), followed by shooting (n=589, 26.85%), action and adventure (AA; n=342, 15.59%), casual (n=302, 13.76%), sports (n=156, 7.11%), sandbox and simulation (SS; n=99, 4.51%), role-playing (n=89, 4.06%), and strategy (n=54, 2.46%) games.

**Table 1:** Descriptive statistics of demographic and gaming characteristics (N=2194).

Variables		Values
Age (years), mean (SD)		14.53 (0.76)
**Sex, n (%)^a^**		
	Men	985 (44.90)
	Women	1174 (53.51)
**Grade level, n (%)**		
	Seventh grade	52 (2.37)
	Eighth grade	969 (44.17)
	Ninth grade	1092 (49.77)
Family economic status^b^, mean (SD)		2.23 (0.70)
Social media time (hours), mean (SD)		18.81 (16.40)
**Number of games played, n (%)**		
	Do not play a game	610 (27.80)
	Playing 1 game	327 (14.90)
	Playing 2 games	525 (23.93)
	Playing 3 games	732 (33.36)
**Number of participants per genre^c^, n (%)**		
	Multiplayer online battle arena	808 (36.83)
	Shooting	589 (26.85)
	Action and adventure	342 (15.59)
	Casual	302 (13.76)
	Sports	156 (7.11)
	Sandbox and simulation	99 (4.51)
	Role-playing	89 (4.06)
	Strategy	54 (2.46)

^a^35 missing values for this variable.

^b^Used a 5-point Likert scale for evaluation (0=extremely poor to 4=excellent).

^c^The number of participants per genre refers to those who reported playing at least one game within that genre.

### Sampling Procedures

Participants were recruited using convenience sampling. A total of 2269 middle school students were approached, of whom 2194 (96.69%) agreed to participate and completed the survey. Self-selection occurred at both the teacher and student levels. At the teacher level, instructors at the tutoring center were invited to assist with data collection, and 10 teachers agreed to participate. At the student level, only students who expressed interest and provided both student assent and parental consent were included. Each participating teacher administered the survey once to each of their classes, all of which were held at the tutoring center. Each participant received ¥10 (US $1.44) in cash as compensation for completing the survey. Study procedures were conducted in compliance with the Declaration of Helsinki and approved by the ethics committee of the first author’s institution (approval number H20250747I). Data were collected between September 10 and October 10, 2025.

### Sample Size, Power, and Precision

We conducted a priori power analysis using G*Power for a linear multiple regression model (fixed model, *R*^2^ increase) to determine the required sample size [58]. We assumed a small effect size for each genre-specific addiction predictor (partial *R*^2^=0.01), an α level of .05, and a desired statistical power of 0.80. The model specified 1 tested predictor and a total of 12 predictors (8 genre-specific addiction variables and 4 covariates). Under these assumptions, the minimum required sample size was 779 participants. Given the achieved sample size of 2194 students, the study was sufficiently powered to detect genre-specific addiction predictors accounting for as little as 1% of unique variance in the outcome.

### Measures and Covariates

Measures and covariates are presented in Textbox 1.

**Measures and covariates.**

1. Genre-specific addiction

The extent to which an individual exhibits excessive and compulsive engagement with a specific genre of video games.

2. Flourishing

A multidimensional state of well-being encompassing domains of happiness and life satisfaction, mental and physical health, meaning and purpose, character and virtue, and close social relationships.

3. Sex

A biological classification of individuals, typically men or women.

4. Social media use

The extent to which students engage with social networking platforms.

5. Family economic status

The financial conditions of a household, reflecting the family’s access to economic resources and its relative position within the socioeconomic hierarchy.

### Data Collection

We contacted a friend of the first author (YL), a physics teacher at the private tutoring center, to inform him about the study’s objectives and data collection procedures. After agreeing to assist, he introduced us to other teachers at the center. Ten additional teachers consented to participate in data collection.

We compiled measurement instruments into a questionnaire, which also included demographic items (age, sex, and grade). Each questionnaire was labeled with a unique identifier number at the bottom of every page to distinguish individual responses. Digital versions of the questionnaire and consent form were sent to the teachers via WeChat (Tencent Holdings Limited). Teachers were instructed to print these materials for use in class.

Each teacher administered the survey once to each of their classes, all of which were held at the tutoring center. At the beginning of each class, teachers explained the study’s purpose and distributed parental consent forms to students who expressed interest in participating. Students who missed 2 classes were excluded from data collection. During the third class session, teachers distributed the questionnaires. Students were told that the survey was anonymous and that neither their parents nor their teachers would have access to their responses. They were instructed to answer honestly, write legibly, and seek clarification if any survey items were unclear. Completing the survey took approximately 10 minutes. The friend of the first author collected all the questionnaires from the teachers and shipped them to the first author. Each teacher received ¥800 (US $115.01) in cash as compensation. The survey questionnaire is available in Multimedia Appendix 1.

### Quality of Measurements

To enhance the quality of measurements, we conducted an online group meeting with all participating teachers before data collection to provide standardized training and explain the data collection protocol in detail. In addition, we trained research assistants in standardized procedures for digitizing paper questionnaires to ensure accurate data entry.

### Instrumentation

#### Video Game Name

The video game name was obtained using the following question: “Please provide the names of all the games you have played during the past month (up to 3). If you have played more than 3, please list the 3 you played most frequently.”

#### Game-Specific Addiction

For each game, participants rated their level of addiction by responding to the following question: “Please indicate your level of addiction for each of the games listed above.” Responses were given on a 5-point Likert scale (0=none, 1=mild, 2=moderate, 3=moderately severe, and 4=severe), with higher scores indicating stronger addiction to a particular game. We used a single item to measure GA for 3 reasons. First, widely used instruments such as the Gaming Addiction Scale for Adolescents [59] and the Internet Gaming Disorder Scale [60] assess overall GA and are therefore unsuitable for measuring game-specific addiction. Second, even if these scales were adapted for game-specific assessment, participants who played 2 or 3 games would need to complete the same scale multiple times, increasing response burden and the risk of response bias. Third, multi-item scales would substantially increase completion time, which was impractical given that the survey was administered during class. Similar single-item measures of GA have been used in prior research, although primarily in adult samples [61,62]. In adolescent populations, single self-diagnostic items assessing other behavioral addictions have shown strong correlations with standardized multi-item instruments [63,64], suggesting that adolescents can comprehend and self-evaluate the severity of behavioral addictions using a single question. The process of transforming game-specific addiction into genre-specific addiction is described in the “Data Processing” section.

#### Flourishing

Flourishing was assessed using the Chinese version of the Human Flourishing Index (HFI) [65]. The scale consists of 10 items covering 5 domains (2 items per domain): (1) happiness and life satisfaction; (2) mental and physical health; (3) meaning and purpose; (4) character and virtue; and (5) close social relationships. Items were rated on an 11-point Likert scale ranging from 0 to 10, with response labels varying across items. Domain scores were computed by summing the scores of the respective items. Overall flourishing was calculated as the sum of all item scores, with higher values reflecting greater flourishing. If any item required to compute a composite score was missing, the corresponding composite score was coded as missing.

#### Covariates

Previous research has found associations between GA, sex [66], social media use [67], and socioeconomic status [68]. To account for their confounding effects [69], we measured participants’ weekly social media time and perceived family economic status using 2 questions. Participants were asked, “In general, how many hours per day do you spend browsing social media platforms (eg, Douyin, Weibo, WeChat Moments, Xiaohongshu, and Bilibili) on weekdays and weekends? Please report to the nearest 0 or 30 minutes (eg, 2 hours 0 minutes, 4 hours 30 minutes).” Total weekly social media time (in hours) was calculated using the formula: 5 × (weekday hours + weekday minutes/60) + 2 × (weekend hours + weekend minutes/60). The family economic status question was, “How would you describe your family’s economic status?” Responses were on a 5-point Likert scale (0=extremely poor to 4=excellent), with higher scores reflecting better family economic status.

### Masking, Psychometrics, Conditions and Design, and Data Processing

As this study employed a cross-sectional observational design without experimental manipulation, masking of participants, data collectors, or outcome assessors was not applicable.

The HFI demonstrated good internal consistency in our sample (Cronbach α=0.93 for overall flourishing, and ranging from 0.76 to 0.89 across domains). Game-specific addiction and family economic status were assessed using single-item measures, and sex and social media use were measured using non–Likert-type items. Therefore, internal consistency coefficients were not applicable for these measures.

This study used a cross-sectional observational design with no experimental manipulation.

Details of the data processing are provided in Multimedia Appendix 2.

### Data Diagnostics

We screened social media use for implausible values and recoded 44 values exceeding 98 hours per week (>14 hours/day) as missing. Extreme outliers were identified using the IQR method. Fourteen values falling below Q1 – 3 × IQR or above Q3 + 3 × IQR were recoded as missing. All other variables were examined for implausible values, and none were identified. Responses were considered missing when participants left an item unanswered. Missing data were handled using multiple imputation by chained equations before statistical analysis. We examined the distributions of the outcome variables and found that overall flourishing and its 5 subscales were negatively skewed across all datasets.

### Analytic Strategy

All statistical analyses were conducted in R (R Foundation) [70]. To reduce the influence of nonnormal outcome distributions on parameter estimation, we employed robust linear regression models [71]. To test the primary hypothesis, for each of the 6 flourishing outcomes, we fitted robust multiple regression models for each dataset, including the 8 genre-specific addiction variables as predictors, and sex, social media time, family economic status, and addiction to other games as covariates. To test the secondary hypothesis, we fitted separate robust regression models for each of the 6 flourishing outcomes with overall GA as a predictor, and sex, social media time, and family economic status as covariates. Parameter estimates were pooled across datasets using Rubin’s rules. We controlled the type I error rate across all models for genre-specific addiction predictors (6 pooled estimates × 8 genres=48 *P* values) and the overall GA predictors (6 pooled estimates) by adjusting the 54 *P* values using the Benjamini-Hochberg false discovery rate procedure. The adjusted *P* values were hereafter referred to as *q* values. Statistical significance for all *q* values was set at α=.05.

As sensitivity analyses, we refitted all models using complete-case data to assess the robustness of the findings to the handling of missing data. The false discovery rate procedure was also applied in complete-case analyses. We further evaluated the robustness of the observed associations to unmeasured confounding by calculating *E* values for statistically significant pooled regression coefficients using the “EValue” package in R [72]. *E* values quantify the minimum strength of association (on the risk ratio scale) that unmeasured confounder(s) would need to have with both the exposure and the outcome, beyond the measured covariates, to fully explain the observed effect [73]. We also calculated *E* values for the CI limit closest to the null, which quantify the minimum strength of association that unmeasured confounder(s) would need to render the observed effect nonsignificant. *E* values were calculated separately for each imputed dataset, and the mean and SD across the 20 imputed datasets were reported.

### Ethical Considerations

This study was approved by the Ethics Committee of Shanghai Jiao Tong University (approval number H20250747I). After the study procedures were explained, students who expressed interest were given parental consent forms to take home. Written informed consent was obtained from parents through signed consent forms before participation. Only students who returned signed parental consent forms and agreed to participate completed the survey, and participation was voluntary. Data were collected anonymously to protect participants’ confidentiality, and only the research team had access to the dataset. Each participant received ¥10 (US $1.44) in cash as compensation for completing the survey. No identifiable images of participants are included in the manuscript or its multimedia appendices.

## Results

### Participant Flow and Recruitment

**Figure 1:**
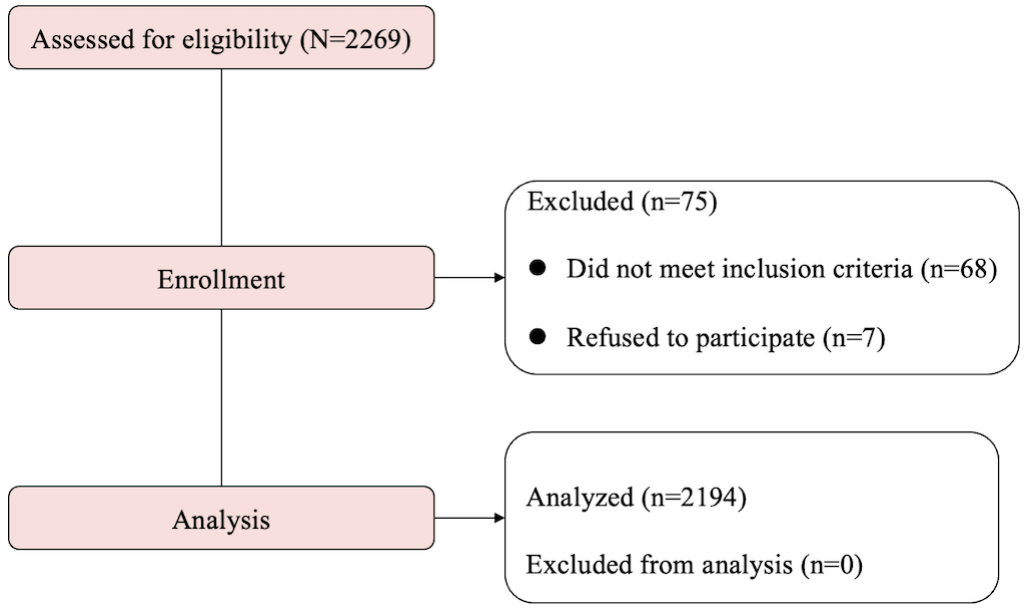
Flow of participants through each stage of the study.

Of the 2269 middle school students eligible for participation, 68 were excluded due to absence from 2 consecutive data collection sessions, and 7 declined to participate. Figure 1 shows the flow of participants across each stage of the study. The final sample included 2194 participants.

Participants were recruited between September 10 and September 24, 2025.

### Statistics and Data Analysis

Inspection of missing data revealed that, among the 2194 participating students, data were missing for age in 22 (1%) cases, sex in 35 (1.60%), grade in 81 (3.69%), family economic status in 254 (11.58%), and social media time in 378 (17.23%). For the HFI, missing data were observed for happiness and life satisfaction in 34 (1.55%) participants, mental and physical health in 34 (1.55%), meaning and purpose in 170 (7.75%), character and virtue in 174 (7.93%), close social relationships in 164 (7.47%), and overall flourishing in 226 (10.30%). Across individual HFI items, missing data ranged from 24 (1.09%) to 164 (7.47%). For genre-specific addiction variables, missing data ranged from 3 (0.14%) to 76 (3.46%). In addition, data were missing for the addiction-to-other-games variable in 24 (1.09%) participants, whereas no missing data were observed for the overall addiction variable.

The Little missing completely at random test applied to the dataset was statistically significant, *χ*^2^_1298_=1495.00 (*P*<.001), indicating that the missing mechanism was not missing completely at random. As several variables exceeded 5% missingness and complete-case analysis can reduce statistical power and introduce bias [74], we assumed that the missingness mechanism was missing at random and applied multiple imputation by chained equations to impute missing data [75]. The predictor matrix included sex, family economic status, social media time, 10 flourishing items, 8 genre-specific addiction variables, and the addiction-to-other-games variable. The overall addiction variable was excluded due to its high linear dependence on other addiction variables. Sex was imputed using logistic regression, and all other variables were imputed using predictive mean matching. We generated 20 imputed datasets, each with 20 iterations. Convergence was confirmed using trace plots, and the distributions of the imputed values aligned well with those of the observed data. After imputation, we calculated composite scores for each dataset.

As shown in Table 2, on average, participants reported a mean overall GA score of 1.49 (SD 1.23). Among genre-specific addictions, participants reported the highest levels of addiction to strategy games (mean 1.65, SD 1.11), followed by AA (mean 1.62, SD 1.24), shooting (mean 1.58, SD 1.21), MOBA (mean 1.46, SD 1.10), SS (mean 1.38, SD 1.36), role-playing (mean 1.36, SD 1.02), casual (mean 1.11, SD 1.09), and sports (mean 0.96, SD 1.01) games. Regarding the HFI, the mean domain scores were 13.54 (SD 4.82) for happiness and life satisfaction, 14.54 (SD 4.72) for mental and physical health, 13.72 (SD 4.58) for meaning and purpose, 12.50 (SD 4.96) for character and virtue, and 14.24 (SD 4.99) for close social relationships. The overall flourishing score averaged 68.60 (SD 20.99).

**Table 2:** Descriptive statistics of gaming addiction and flourishing outcomes (N=2194).

Variables		Values, mean (SD)
Overall addiction		1.49 (1.23)
**Gaming addiction per genre**		
	Multiplayer online battle arena	1.46 (1.10)
	Shooting	1.58 (1.21)
	Action and adventure	1.62 (1.24)
	Casual	1.11 (1.09)
	Sports	0.96 (1.01)
	Sandbox and simulation	1.38 (1.36)
	Role-playing	1.36 (1.02)
	Strategy	1.65 (1.11)
**Human Flourishing Index**		
	Happiness and life satisfaction	13.54 (4.82)
	Mental and physical health	14.54 (4.72)
	Meaning and purpose	13.72 (4.58)
	Character and virtue	12.50 (4.96)
	Close social relationships	14.24 (4.99)
	Overall flourishing	68.60 (20.99)

**Figure 2:**
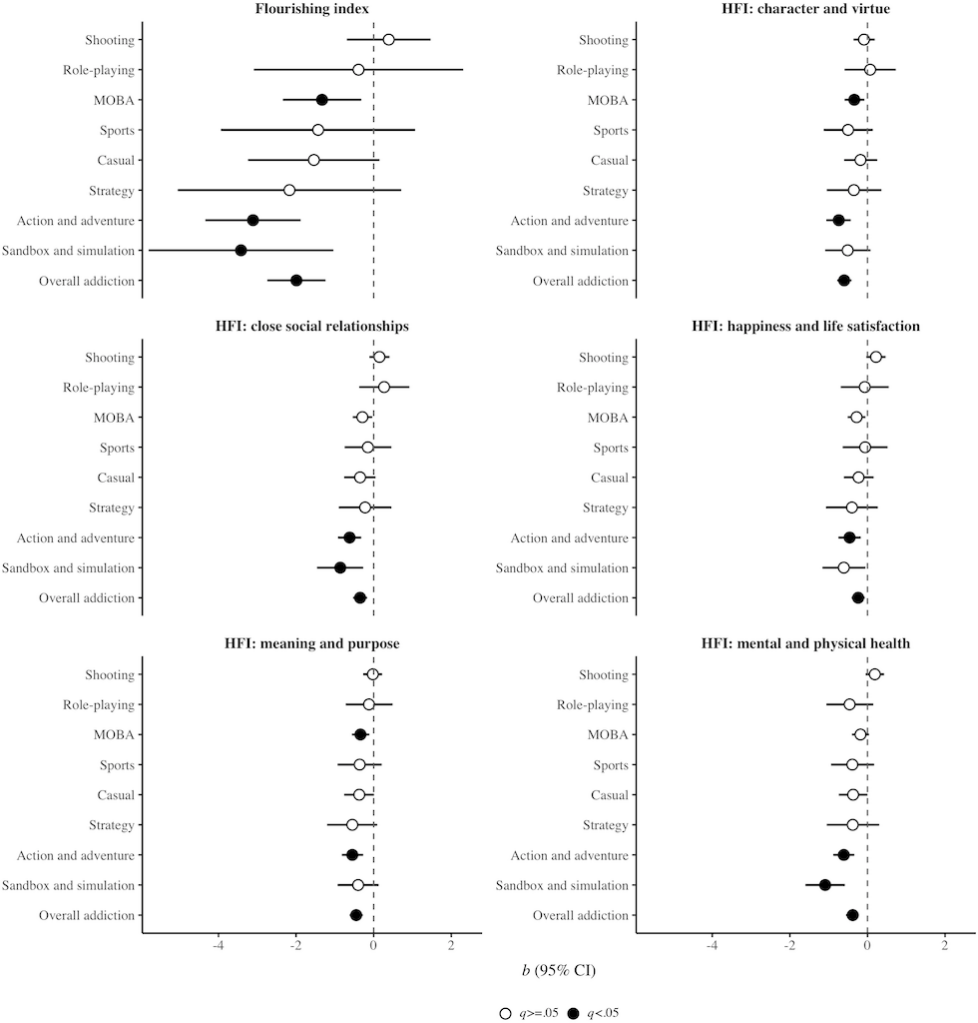
Robust linear regression results showing the associations between gaming addiction and flourishing outcomes based on multiple imputed data (N=2194). HFI: Human Flourishing Index; MOBA: multiplayer online battle arena.

Table 3 and Figure 2 present robust linear regression results examining the associations between GA and flourishing outcomes using multiply imputed data. Regarding the primary hypothesis, AA addiction was the only predictor that was negatively associated with all flourishing outcomes (overall flourishing: *b*=–3.11, 95% CI –4.34 to –1.88, *q*<.001; happiness and life satisfaction: *b*=–0.46, 95% CI –0.75 to –0.17, *q*=.009; mental and physical health: *b*=–0.61, 95% CI –0.88 to –0.34, *q*<.001; meaning and purpose: *b*=–0.55, 95% CI –0.82 to –0.27, *q*=.001; character and virtue: *b*=–0.74, 95% CI –1.06 to –0.43, *q*<.001; and close social relationships: *b*=–0.62, 95% CI –0.92 to –0.32, *q*<.001). MOBA addiction was negatively associated with overall flourishing (*b*=–1.33, 95% CI –2.34 to –0.32, *q*=.03), meaning and purpose (*b*=–0.34, 95% CI –0.56 to –0.11, *q*=.02), and character and virtue (*b*=–0.34, 95% CI –0.59 to –0.08, *q*=.03). SS addiction was negatively associated with overall flourishing (*b*=–3.42, 95% CI –5.80 to –1.04, *q*=.02), mental and physical health (*b*=–1.09, 95% CI –1.60 to –0.58, *q*<.001), and close social relationships (*b*=–0.86, 95% CI –1.46 to –0.27, *q*=.02). Other genre-specific associations were not statistically significant (*q*≥.05 for all; discussed later). Analyses testing the secondary hypothesis showed that overall GA was negatively associated with all flourishing outcomes (overall flourishing: *b*=–1.99, 95% CI –2.74 to –1.24, *q*<.001; happiness and life satisfaction: *b*=–0.24, 95% CI –0.41 to –0.07, *q*=.02; mental and physical health: *b*=–0.38, 95% CI –0.55 to –0.22, *q*<.001; meaning and purpose: *b*=–0.45, 95% CI –0.62 to –0.28, *q*<.001; character and virtue: *b*=–0.60, 95% CI –0.78 to –0.41, *q*<.001; and close social relationships: *b*=–0.35, 95% CI –0.53 to –0.17, *q*=.001).

**Table 3:** Robust linear regression results for the associations between gaming addiction and flourishing outcomes based on multiple imputed data (N=2194).

Variables^a^				Multiple imputation analysis^b^		
				*b* (95% CI)		*q* value^c^
**Outcome: overall flourishing**						
	**Predictors (genre-specific model)**					
		Multiplayer online battle arena	–1.33 (–2.34 to –0.32)		.03	
		Shooting	0.39 (–0.69 to 1.47)		.57	
		Casual	–1.54 (–3.24 to 0.15)		.17	
		Action and adventure	–3.11 (–4.34 to –1.88)		<.001	
		Sandbox and simulation	–3.42 (–5.80 to –1.04)		.02	
		Sports	–1.43 (–3.94 to 1.07)		.36	
		Strategy	–2.17 (–5.05 to 0.71)		.22	
		Role-playing	–0.39 (–3.09 to 2.31)		.84	
	**Predictor (overall addiction model)**					
		Overall addiction	–1.99 (–2.74 to –1.24)		<.001	
**Outcome: happiness and life satisfaction**						
	**Predictors (genre-specific model)**					
		Multiplayer online battle arena	–0.28 (–0.51 to –0.05)		.05	
		Shooting	0.22 (–0.03 to 0.47)		.18	
		Casual	–0.23 (–0.61 to 0.16)		.36	
		Action and adventure	–0.46 (–0.75 to –0.17)		.009	
		Sandbox and simulation	–0.61 (–1.16 to –0.05)		.08	
		Sports	–0.06 (–0.64 to 0.52)		.84	
		Strategy	–0.40 (–1.07 to 0.27)		.36	
		Role-playing	–0.07 (–0.69 to 0.55)		.84	
	**Predictor (overall addiction model)**					
		Overall addiction	–0.24 (–0.41 to –0.07)		.02	
**Outcome: mental and physical health**						
	**Predictors (genre-specific model)**					
		Multiplayer online battle arena	–0.18 (–0.40 to 0.04)		.20	
		Shooting	0.19 (–0.05 to 0.43)		.20	
		Casual	–0.37 (–0.74 to 0.00)		.12	
		Action and adventure	–0.61 (–0.88 to –0.34)		<.001	
		Sandbox and simulation	–1.09 (–1.60 to –0.58)		<.001	
		Sports	–0.39 (–0.94 to 0.17)		.26	
		Strategy	–0.38 (–1.05 to 0.30)		.36	
		Role-playing	–0.46 (–1.06 to 0.15)		.22	
	**Predictor (overall addiction model)**					
		Overall addiction	–0.38 (–0.55 to –0.22)		<.001	
**Outcome: meaning and purpose**						
	**Predictors (genre-specific model)**					
		Multiplayer online battle arena	–0.34 (–0.56 to –0.11)		.02	
		Shooting	–0.02 (–0.27 to 0.22)		.84	
		Casual	–0.37 (–0.76 to 0.01)		.13	
		Action and adventure	–0.55 (–0.82 to –0.27)		.001	
		Sandbox and simulation	–0.40 (–0.93 to 0.13)		.22	
		Sports	–0.36 (–0.93 to 0.21)		.32	
		Strategy	–0.55 (–1.20 to 0.09)		.18	
		Role-playing	–0.12 (–0.72 to 0.49)		.78	
	**Predictor (overall addiction model)**					
		Overall addiction	–0.45 (–0.62 to –0.28)		<.001	
**Outcome: character and virtue**						
	**Predictors (genre-specific model)**					
		Multiplayer online battle arena	–0.34 (–0.59 to –0.08)		.03	
		Shooting	–0.09 (–0.36 to 0.19)		.61	
		Casual	–0.18 (–0.60 to 0.25)		.51	
		Action and adventure	–0.74 (–1.06 to –0.43)		<.001	
		Sandbox and simulation	–0.51 (–1.09 to 0.08)		.18	
		Sports	–0.50 (–1.13 to 0.14)		.22	
		Strategy	–0.35 (–1.05 to 0.36)		.43	
		Role-playing	0.07 (–0.59 to 0.73)		.84	
	**Predictor (overall addiction model)**					
		Overall addiction	–0.60 (–0.78 to –0.41)		<.001	
**Outcome: close social relationships**						
	**Predictors (genre-specific model)**					
		Multiplayer online battle arena	–0.29 (–0.54 to –0.04)		.07	
		Shooting	0.15 (–0.11 to 0.41)		.36	
		Casual	–0.35 (–0.76 to 0.05)		.18	
		Action and adventure	–0.62 (–0.92 to –0.32)		<.001	
		Sandbox and simulation	–0.86 (–1.46 to –0.27)		.02	
		Sports	–0.15 (–0.75 to 0.46)		.72	
		Strategy	–0.22 (–0.90 to 0.46)		.61	
		Role-playing	0.27 (–0.37 to 0.92)		.51	
	**Predictor (overall addiction model)**					
		Overall addiction	–0.35 (–0.53 to –0.17)		.001	

^a^Game genres denote addiction to the corresponding genres.

^b^All models included sex, family economic status, and social media time as covariates; genre-specific models additionally controlled for addiction-to-other-games.

^c^*q* indicates that raw *P* values were adjusted using the Benjamini-Hochberg false discovery rate procedure.

Although overall GA was negatively associated with all flourishing domains, the genre-specific findings indicate substantial heterogeneity in the addiction-flourishing relationship across game genres. The most prominent genre-specific predictor was AA addiction, which was associated with lower scores across all flourishing domains, suggesting that adolescents with AA addiction may constitute a particularly high-risk subgroup with broadly impaired well-being. Although both SS and MOBA addiction were associated with lower overall flourishing, their domain-specific patterns differed. The SS-flourishing association appeared to be driven primarily by poorer mental and physical health and weaker close social relationships, whereas the MOBA-flourishing association was driven mainly by lower meaning and purpose and character and virtue. The absence of significant associations for other genres (overall flourishing: *q*=.57, .17, .36, .22, and .84; happiness and life satisfaction: *q*=.18, .36, .84, .36, and .84; mental and physical health: *q*=.20, .12, .26, .36, and .22; meaning and purpose: *q*=.84, .13, .32, .18, and .78; character and virtue: *q*=.61, .51, .22, .43, and .84; and close social relationships: *q*=.36, .18, .72, .61, and .51, for shooting, casual, sports, strategy, and role-playing, respectively) suggests that addiction to these genres may be comparatively less detrimental to flourishing than addiction to AA, SS, and MOBA.

We conducted model diagnostics to evaluate regression assumptions. Multicollinearity among predictors was assessed using variance inflation factors. The maximal variance inflation factor across the 20 imputed datasets and all outcome variables (including the overall GA models) was 1.22, indicating no problematic collinearity among predictors [76]. Visual inspection of residuals-versus-fitted plots across all imputed datasets and models indicated no substantial departures from linearity or other patterns suggesting model misspecification.

Multimedia Appendix 3 presents the results of the robust linear regression analyses examining the associations between GA and flourishing outcomes using complete-case data. The pattern of significant predictors was consistent with the analyses based on multiple imputed data for overall flourishing, mental and physical health, meaning and purpose, and character and virtue. Compared with the multiple imputation analyses, the effect of overall GA on happiness and life satisfaction became nonsignificant in the complete-case analysis (*b*=–0.22, 95% CI –0.41 to –0.03, *q*=.07), whereas the effect of MOBA on close social relationships became statistically significant in the complete-case analysis (*b*=–0.39, 95% CI –0.66 to –0.12, *q*=.02). Despite these discrepancies, the overall pattern of results was generally consistent across the 2 approaches, suggesting that the findings were largely robust to the handling of missing data.

Multimedia Appendix 4 presents the *E* values for the significant pooled effects from the robust linear regression analyses based on multiple imputed data. Across all flourishing outcomes, the *E* values for the regression coefficients ranged from 1.31 to 1.55, indicating moderate robustness to unmeasured confounding. This suggests that unmeasured confounder(s) would need to be associated with both GA and flourishing outcomes by at least a 1.3-fold risk ratio, beyond the measured covariates, to fully explain the observed effects. The *E* values for the CI limits closest to the null ranged from 1.14 to 1.43, with 7 values below 1.20. These findings indicate that relatively weak unmeasured confounder(s) could attenuate the statistical significance of some estimates. Therefore, certain genre-specific associations should be interpreted with caution, given their potential susceptibility to residual confounding.

## Discussion

### Principal Findings

Using VanderWeele’s multidimensional flourishing framework, this study examined genre-specific associations between GA and flourishing among adolescents. Consistent with our primary hypothesis, the associations between GA and flourishing varied across game genres rather than being uniform. Addiction to AA, SS, and MOBA games showed significant negative associations with all or some flourishing outcomes, whereas addiction to other genres was not significantly associated with any flourishing outcomes. Consistent with our secondary hypothesis, overall GA was negatively associated with all flourishing outcomes.

### Interpretations and Comparisons to Existing Literature

A substantial body of research has linked adolescents’ GA to a range of health outcomes, including reduced life satisfaction [7], poorer health [7,17], lower meaning in life [35], lower empathy and moral levels [38,39], and difficulties in social relationships [14,26]. Consistent with this literature, this study found that overall GA was negatively associated with all 5 flourishing subdomains (ie, happiness and life satisfaction, mental and physical health, meaning and purpose, character and virtue, and close social relationships). In addition, our findings extend prior work by showing that the negative correlates of GA are not confined to isolated facets of well-being but instead span the full spectrum of adolescents’ flourishing. This suggests that addictive gaming may constrain adolescents’ capacity to thrive across multiple life domains. Moreover, our results indicate that the adverse association between GA and flourishing is not uniform across game genres. While addiction to certain genres was negatively related to flourishing, addiction to others was not associated with any flourishing outcomes.

The genre-specific analyses revealed that AA addiction was the only consistent negative predictor of overall flourishing and all 5 of its domains. Several mechanisms may explain these findings. First, AA games possess design features that may heighten the risk of reduced flourishing. Compared with other genres, AA games often feature open-world exploration and story-driven adventures that lack natural stopping points [77]. Such features can promote prolonged, uninterrupted play, frequently in solitary contexts, which may displace real-life activities essential for flourishing [78,79]. Second, AA games commonly include fast-paced, skill-based activities (eg, defeating enemies and solving puzzles) that produce rapid cycles of in-game achievement [80]. Although such virtual accomplishments may temporarily “scratch the itch” for success [81], prior research suggests that they function more as compensators than as substitutes for real-world competence [82]. Thus, adolescents with addictive gaming patterns may feel competent during gameplay, yet their underlying need for real-world competence remains unmet, potentially leading to reduced well-being [83]. Third, adolescents with preexisting low levels of flourishing may be particularly drawn to AA games. Those with low life satisfaction, weak social relationships, poor emotional well-being, or a lack of life purpose may find AA games especially appealing because they offer immersive narratives, clear missions, and rapid reward cycles that provide a temporary sense of escape, competence, and progression [84]. When real life feels unsatisfying, completing quests, advancing storylines, and gaining in-game achievements can serve as substitutes for the meaning and accomplishments they struggle to experience in daily life, making these games particularly attractive [85].

Higher MOBA addiction was associated with poorer character and virtue. This relationship may be explained by the highly competitive and performance-driven nature of these games, which emphasize winning, dominance, and skill mastery rather than empathy or moral reflection [86]. Prior research has shown that many MOBA players experience in-game hostility and toxic communication [87], which may erode prosocial tendencies and empathy—core elements of character and virtue. Higher MOBA addiction was also associated with a lower sense of meaning and purpose. Similar to AA games, MOBA games provide clear goals (eg, destroying the enemy nexus) and immediate rewards (eg, rank increases after winning) [88], which are often more attainable than real-life achievements. When adolescents repeatedly turn to these games for accomplishment, real-world goals such as academic progress or personal growth may feel less rewarding or more difficult to pursue [82], leading to fewer meaningful experiences and a weakened sense of purpose [89]. It is also plausible that adolescents who already exhibit lower character and virtue or a reduced sense of meaning and purpose are more vulnerable to MOBA addiction, as these game-design features may temporarily compensate for these deficits [90].

Higher SS addiction was associated with fewer close social relationships and poorer mental and physical health. Several mechanisms may account for these associations. First, SS games are typically structured around solitary play, allowing players to advance independently, in contrast to team-based genres that require interpersonal coordination [91]. Such solitary engagement may displace real-world interaction, which is essential for building and maintaining close social relationships [92]. Second, SS games provide a strong sense of autonomy and control (eg, players can design cities, manage resources, and make strategic decisions about budgets and policies) [93], which may be particularly appealing to adolescents who feel powerless in real life due to limited social support [94,95]. Third, adolescents with poorer mental health resulting from real-world challenges may be prone to playing these games, as they allow them to simulate idealized achievements and life scenarios (eg, building perfect cities, running successful businesses, or creating ideal families) [96]. These experiences may temporarily compensate for dissatisfaction with real-life circumstances [82].

The absence of associations between addiction to shooting, sports, casual, strategy, or role-playing games and flourishing may be explained by several factors. First, these genres are highly heterogeneous in their modes of play [97-100]. For example, shooting games may involve competitive player-versus-player matches, cooperative missions against computer-controlled opponents, or single-player narrative progression [100]. Sports games can be played through team-on-team matches or career modes that simulate long-term athlete development [100]. Casual, strategy, and role-playing games can be played in either solitary or highly social modes [97-99]. This variability means that potential positive and negative associations with flourishing at the game level may offset one another at the genre level. Second, many games in these genres feature short matches or rounds, which may make disengagement easier compared with genres that lack natural stopping points [101]. This, in turn, reduces the likelihood of prolonged immersion that displaces real-life activities essential for flourishing [102,103].

### Implications

The findings of this study have several practical implications. First, higher addiction to shooting, sports, casual, strategy, or role-playing games was not associated with poorer flourishing outcomes. This suggests that encouraging adolescents to spend their gaming time within these relatively lower-risk genres may not increase the likelihood of adverse outcomes associated with GA. This does not imply that addiction to such games is harmless. Rather, when gaming is already part of adolescents’ lives, guiding them toward lower-risk genres may represent a more realistic harm-reduction strategy. Second, when adolescents are highly engaged in AA, MOBA, or SS games, targeted strategies may be required to reduce addiction-related risks. For parents, setting clear time limits and enforcing mandatory breaks may help prevent prolonged, immersive play. Conversations that unpack why certain games feel particularly absorbing may also increase adolescents’ awareness of risky game design features, thereby empowering them to better self-regulate their play. Providing alternative activities that offer similar psychological rewards may also be effective. For example, adolescents drawn to SS games may find comparable satisfaction in robotics clubs, art workshops, or sandbox-style board games, which can fulfill needs for autonomy and creativity. MOBA players may benefit from real-world team sports, which can satisfy desires for competition, collaboration, and skill mastery. Third, for policy makers, rather than imposing one-size-fits-all rules, policies could be more targeted toward high-risk game genres. For instance, China’s current “Online Game Anti-Indulgence System” restricts adolescents to a single hour of play on weekend evenings across all games (although many adolescents have learned ways to circumvent this rule). A more nuanced policy could impose stricter limits on high-risk genres while allowing more time for genres associated with fewer negative health outcomes. Such differentiation could better balance adolescents’ enjoyment of gaming with the goal of minimizing potential harm to their well-being.

### Limitations

Several limitations should be considered when interpreting the findings. First, the results may be influenced by the genre classification approach adopted in this study. Although the categorization was informed by established frameworks, substantial variation may still exist among games within the same genre with respect to design features that could lead to distinct associations with health-related outcomes. Future research would benefit from developing or validating a genre classification system specifically optimized for health research. Such a system could reduce within-genre heterogeneity and facilitate more robust conclusions regarding genre-specific associations with adolescent health. Second, the cross-sectional design of this study limits causal inference between GA and flourishing. The *E* value analysis also suggested that some associations could potentially be explained by unmeasured confounders of small magnitude. To strengthen causal inference, future studies could employ longitudinal and experimental designs to clarify whether genre-specific GA undermines flourishing, whether adolescents with lower flourishing are more likely to develop addiction to particular genres, or whether these processes operate reciprocally. Third, due to practical constraints, GA was measured using single-item self-reports, which are inherently less precise than multi-item scales and may have introduced measurement error. Future studies should use validated multi-item measures when feasible.

### Conclusions

This study provides novel evidence that the association between GA and adolescent flourishing varies by game genre. In contrast to prior work that treats health as a narrow or unidimensional construct, the adoption of a multidimensional health framework offers a more nuanced understanding of gaming-related risks. The findings contribute to the field by indicating that addiction to AA, MOBA, and SS games poses greater risks to adolescent health than addiction to other genres. Accordingly, prevention, education, and policy efforts should prioritize these higher-risk genres to more effectively support adolescent health.

## Appendix

Multimedia Appendix 1 Survey questionnaire.

Multimedia Appendix 2 Data processing.

Multimedia Appendix 3 Results of the robust linear regression analyses examining the associations between gaming addiction and flourishing outcomes using complete-case data.

Multimedia Appendix 4 E values for the significant pooled effects from the robust linear regression analyses based on multiple imputed data.

## Abbreviations

AA: action and adventure
GA: gaming addiction
HFI: Human Flourishing Index
JARS-Quant: Journal Article Reporting Standards for Quantitative Research
MOBA: multiplayer online battle arena
SS: sandbox and simulation

## Data Availability

The datasets generated or analyzed during this study are not publicly available because they will be used for secondary analyses in other ongoing studies, but are available from the corresponding author (YL) upon reasonable request.
