# Supplementary material for: Genre-Specific Gaming Addiction and Flourishing in Adolescents: Cross-Sectional Survey Study
Source: J Med Internet Res. 2026 Feb 12;28:e89319. doi: 10.2196/89319 (PMC12946777; doi:10.2196/89319)
Supplement: Multimedia Appendix 1 [file jmir_v28i1e89319_app1.docx]

**欢迎参加本研究！**

1.你的年龄： 2.你的性别：○男 ○女 3.你的年级：○初一 ○初二 ○初三

4.你觉得你的家庭经济状况如何？○特别不好 ○不太好 ○一般 ○比较好 ○非常好

问题5-6与游戏行为有关，若你在过去的1个月没有玩过游戏可跳过这些题，从第7题继续作答。

5.请填写你在**过去1个月**玩过的所有游戏的名称（最多填写三款，若超过三款，请填写最经常玩的三款游戏）。

|  | 名称 |
| --- | --- |
| 游戏1 |  |
| 游戏2 |  |
| 游戏3 |  |

6.请勾选自己对上述每款游戏的上瘾程度：

|  | 没有 | 很轻 | 中等 | 偏重 | 严重 |
| --- | --- | --- | --- | --- | --- |
| 游戏1 | ○ | ○ | ○ | ○ | ○ |
| 游戏2 | ○ | ○ | ○ | ○ | ○ |
| 游戏3 | ○ | ○ | ○ | ○ | ○ |

7.一般来说，在周一至周五、周六和周日，你**平均每天**浏览社交媒体（如抖音、微博、朋友圈、小红书、b站等）的时间为（精确至0或30分，例如：2小时0分、4小时30分）？

周一至周五： 小时 分；周六和周日： 小时 分。

8.总的来说，你对最近一段时间生活的满意度如何？

完全不满意 完全满意

| 0 | 1 | 2 | 3 | 4 | 5 | 6 | 7 | 8 | 9 | 10 |
| --- | --- | --- | --- | --- | --- | --- | --- | --- | --- | --- |

9.整体而言，你觉得自己过得开心吗？

非常不开心 非常开心

| 0 | 1 | 2 | 3 | 4 | 5 | 6 | 7 | 8 | 9 | 10 |
| --- | --- | --- | --- | --- | --- | --- | --- | --- | --- | --- |

10.整体而言，你如何评价自己的身体健康状况？

非常差 非常好

| 0 | 1 | 2 | 3 | 4 | 5 | 6 | 7 | 8 | 9 | 10 |
| --- | --- | --- | --- | --- | --- | --- | --- | --- | --- | --- |

11.整体而言，你如何评价自己的心理健康状况？

非常差 非常好

| 0 | 1 | 2 | 3 | 4 | 5 | 6 | 7 | 8 | 9 | 10 |
| --- | --- | --- | --- | --- | --- | --- | --- | --- | --- | --- |

12.整体而言，你觉得在生活中所做的事情有多大程度是值得的？

完全不值得 完全值得

| 0 | 1 | 2 | 3 | 4 | 5 | 6 | 7 | 8 | 9 | 10 |
| --- | --- | --- | --- | --- | --- | --- | --- | --- | --- | --- |

13.我了解我人生的意义。

非常不同意 非常同意

| 0 | 1 | 2 | 3 | 4 | 5 | 6 | 7 | 8 | 9 | 10 |
| --- | --- | --- | --- | --- | --- | --- | --- | --- | --- | --- |

14.我在各种情况下都努力做好事，即便在艰难时刻也是如此。

非常不同意 非常同意

| 0 | 1 | 2 | 3 | 4 | 5 | 6 | 7 | 8 | 9 | 10 |
| --- | --- | --- | --- | --- | --- | --- | --- | --- | --- | --- |

15.我总是能为了以后更大的幸福而放弃一些当下的快乐。

非常不同意 非常同意

| 0 | 1 | 2 | 3 | 4 | 5 | 6 | 7 | 8 | 9 | 10 |
| --- | --- | --- | --- | --- | --- | --- | --- | --- | --- | --- |

16.我对自己的友谊和人际关系感到满意。

非常不同意 非常同意

| 0 | 1 | 2 | 3 | 4 | 5 | 6 | 7 | 8 | 9 | 10 |
| --- | --- | --- | --- | --- | --- | --- | --- | --- | --- | --- |

17.我的人际关系达到了我所期望的满意程度。

非常不同意 非常同意

| 0 | 1 | 2 | 3 | 4 | 5 | 6 | 7 | 8 | 9 | 10 |
| --- | --- | --- | --- | --- | --- | --- | --- | --- | --- | --- |
