# Supplementary material for: Genre-Specific Gaming Addiction and Flourishing in Adolescents: Cross-Sectional Survey Study
Source: J Med Internet Res. 2026 Feb 12;28:e89319. doi: 10.2196/89319 (PMC12946777; doi:10.2196/89319)
Supplement: Multimedia Appendix 2 [file jmir_v28i1e89319_app2.docx]

*Data Processing*

Upon receiving the paper questionnaires, we scanned them using a Fujitsu IX1400 scanner (Kawasaki, Japan). In total, 2194 paper questionnaires (some with missing pages) were scanned, indicating that 3.3% (75/2269) of students either declined to participate or were absent for two consecutive data collection classes. The paper versions were then destroyed using a shredder. To prepare the data for analysis, we created an identical online version of the questionnaire in Wen Juan Xing (Ranxing Information Technology Co., Ltd., China). Six undergraduate research assistants manually entered the scanned questionnaires into the online questionnaire.

Before data entry, we held a face-to-face training session with the research assistants. They were instructed to enter responses exactly as written, including suspicious patterns (e.g., selecting the same option for all items in the HFI), and to keep blank items blank. If a questionnaire was missing pages, data from available pages should be entered, and data on missing pages were left empty. Assistants were instructed to use specific codes to document problematic data: 999 = illegible response; 888 = invalid information (e.g., name written in place of age); 777 = multiple answers for a single-choice item; 666 = ambiguous response (e.g., check mark between two options); 555 = other problems. Digitization lasted approximately one month. Each assistant received ¥800 (US $115.01) as compensation. After downloading the dataset from Wen Juan Xing, we identified 22 duplicated entries using questionnaire ID numbers. Following the removal of duplicates, the dataset contained 2169 unique questionnaires. The 25 missing questionnaires were entered into the dataset by cross-checking the questionnaire ID numbers between the scanned and digital questionnaires. Data quality was assessed by inspecting the frequency of problematic codes (25 instances of 999, 43 of 888, 31 of 777, 4 of 666, and none of 555). The low frequency of these entries indicated good data quality, and all such entries were recoded as missing for subsequent analyses.

We calculated the frequencies of all reported video game names. In total, 324 unique game names were reported, with frequencies ranging from 1 to 699. Of these, 215 games were reported only once, and 109 appeared more than once. For games reported more than once, game names that differed from the official game names due to abbreviations, capitalization, or minor spelling errors were recoded to the official game names. This procedure was not applied to single-entry games, as the intended meaning for some could not be cross-validated from other participants. In addition, because of the limited availability of online information for many of these games, verifying their genres for subsequent analyses was difficult.

Four research assistants categorized all games reported more than once into eight genres: action and adventure (AA), sandbox and simulation (SS), multiplayer online battle arena (MOBA), shooting, strategy, casual, sports, and role-playing. Games that did not fit into any of these genres were categorized as “others.” These genres were created based on established genre frameworks from previous studies [1–4]. Two assistants independently categorized 54 identical games, while the other two categorized the remaining 55 games. Each assistant independently assigned games to genres using information obtained from online game forums and gameplay videos. The first author compared the categorizations of same games, and discrepancies were resolved through discussion with the assistants. When a game could be assigned to multiple genres, it was classified to the genre that best represented its primary gameplay characteristics. After classification, there were 18 AA games, 2 SS games, 7 MOBA games, 16 shooting games, 8 strategy games, 22 casual games, 8 sports games, 20 role-playing games, and 8 other games.

We created genre-specific addiction variables for each game genre. For participants who reported not playing any games, all genre-specific addiction scores were coded as 0. If all the reported games did not belong to a given genre, the addiction score for that genre was set to 0. When a participant reported one game within a genre, the corresponding addiction score of that game was used for that genre. If that score was missing, the genre score was coded as missing. For participants who reported two or three games within the same genre, the genre-specific addiction score was defined as the highest addiction score among those games. In cases with missing scores, available scores were used according to the following rule: (1) if not all scores were missing, the remaining score(s) determined the genre-specific score; (2) if all were missing, the genre-specific score was coded as missing. We chose to use the highest score rather than the mean or sum because averaging would underestimate a participant’s true level of addiction, whereas summing would yield an impossible severity of addiction. For example, if a participant reported addiction scores of 2 and 4 for two games within the same genre, the mean (3) would downplay the severity reflected by the score of 4, and the sum (6) would exceed the most possible severity score of 4. Thus, using the highest score provided a more realistic reflection of a participant’s genre-specific GA. An addiction-to-other-games variable was created using the same rules, based on addiction scores from the eight games categorized as “others” and from games reported only once. This variable allowed us to examine the associations between genre-specific addiction and flourishing while controlling for addiction to other games. In addition, an overall GA variable was created, defined as each participant’s highest score across all genre-specific addiction variables and the addiction-to-other-games variable.

**References**

1. Laconi S, Pirès S, Chabrol H. Internet gaming disorder, motives, game genres and psychopathology. *Computers in Human Behavior*. 2017;75:652-659.

2. Na E, Choi I, Lee TH, et al. The influence of game genre on Internet gaming disorder. *J Behav Addict*. 2017;6(2):248-255. doi:10.1556/2006.6.2017.033

3. Oka T, Kubo T, Murakami M, Kobayashi N. The relationship of game genres, in-game purchases, and playing duration with probable gaming disorder in two independent, large-scale online surveys of Japanese adults. *JBA*. 2024;13(1):205-214. doi:10.1556/2006.2023.00076

4. Ye X, Fong TC, Yip PS. A cross-lagged panel network model on internet gaming disorder and depressive symptoms concerning preferences for game genres. *Journal of Affective Disorders*. 2025;375:27-34.
