# Supplementary material for: Genre-Specific Gaming Addiction and Flourishing in Adolescents: Cross-Sectional Survey Study
Source: J Med Internet Res. 2026 Feb 12;28:e89319. doi: 10.2196/89319 (PMC12946777; doi:10.2196/89319)
Supplement: Multimedia Appendix 3 [file jmir_v28i1e89319_app3.docx]

**Table S1.** Robust linear regression results for the associations between gaming addiction and flourishing outcomes using complete-case data.

| Variables^a^ | Complete-Case Analysis^b^ | |
| --- | --- | --- |
|  | *b* (95% CI) | *q* value^c^ |
| Outcome: Overall flourishing (N=1490) |  |  |
| Predictors (genre-specific model) |  |  |
| Multiplayer online battle arena | -1.39 (-2.51 to -0.28) | .045 |
| Shooting | 0.14 (-1.06 to 1.35) | .90 |
| Casual | -1.54 (-3.40 to 0.32) | .23 |
| Action and adventure | -2.76 (-4.17 to -1.36) | .001 |
| Sandbox and simulation | -4.12 (-6.92 to -1.31) | .02 |
| Sports | -1.00 (-3.68 to 1.68) | .61 |
| Strategy | -0.91 (-4.10 to 2.28) | .68 |
| Role-playing | -0.17 (-2.82 to 2.48) | .95 |
| Predictor (overall addiction model) |  |  |
| Overall addiction | -1.74 (-2.59 to -0.90) | .001 |
| Outcome: Happiness and life satisfaction (N=1555) |  |  |
| Predictors (genre-specific model) |  |  |
| Multiplayer online battle arena | -0.26 (-0.51 to -0.01) | .12 |
| Shooting | 0.17 (-0.10 to 0.44) | .41 |
| Casual | -0.22 (-0.64 to 0.20) | .48 |
| Action and adventure | -0.40 (-0.72 to -0.08) | .045 |
| Sandbox and simulation | -0.64 (-1.30 to 0.03) | .15 |
| Sports | -0.07 (-0.69 to 0.55) | .90 |
| Strategy | -0.42 (-1.09 to 0.26) | .41 |
| Role-playing | -0.03 (-0.63 to 0.58) | .96 |
| Predictor (overall addiction model) |  |  |
| Overall addiction | -0.22 (-0.41 to -0.03) | .07 |
| Outcome: Mental and physical health (N=1557) |  |  |
| Predictors (genre-specific model) |  |  |
| Multiplayer online battle arena | -0.09 (-0.33 to 0.15) | .61 |
| Shooting | 0.14 (-0.12 to 0.41) | .48 |
| Casual | -0.36 (-0.77 to 0.04) | .18 |
| Action and adventure | -0.68 (-0.98 to -0.37) | <.001 |
| Sandbox and simulation | -1.08 (-1.67 to -0.49) | .002 |
| Sports | -0.43 (-1.02 to 0.17) | .33 |
| Strategy | 0.02 (-0.69 to 0.74) | .96 |
| Role-playing | -0.37 (-0.97 to 0.22) | .41 |
| Predictor (overall addiction model) |  |  |
| Overall addiction | -0.33 (-0.52 to -0.14) | .004 |
| Outcome: Meaning and purpose (N=1526) |  |  |
| Predictors (genre-specific model) |  |  |
| Multiplayer online battle arena | -0.33 (-0.58 to -0.09) | .03 |
| Shooting | -0.07 (-0.33 to 0.20) | .72 |
| Casual | -0.40 (-0.81 to 0.02) | .15 |
| Action and adventure | -0.46 (-0.77 to -0.16) | .02 |
| Sandbox and simulation | -0.32 (-0.95 to 0.31) | .48 |
| Sports | -0.31 (-0.91 to 0.29) | .48 |
| Strategy | -0.35 (-1.03 to 0.32) | .48 |
| Role-playing | -0.11 (-0.70 to 0.48) | .80 |
| Predictor (overall addiction model) |  |  |
| Overall addiction | -0.41 (-0.60 to -0.22) | <.001 |
| Outcome: Character and virtue (N=1522) |  |  |
| Predictors (genre-specific model) |  |  |
| Multiplayer online battle arena | -0.37 (-0.64 to -0.10) | .03 |
| Shooting | -0.08 (-0.37 to 0.20) | .68 |
| Casual | -0.20 (-0.66 to 0.25) | .54 |
| Action and adventure | -0.67 (-1.01 to -0.33) | .001 |
| Sandbox and simulation | -0.48 (-1.15 to 0.18) | .32 |
| Sports | -0.20 (-0.86 to 0.46) | .67 |
| Strategy | -0.26 (-0.96 to 0.44) | .61 |
| Role-playing | 0.00 (-0.64 to 0.65) | >.99 |
| Predictor (overall addiction model) |  |  |
| Overall addiction | -0.54 (-0.74 to -0.34) | <.001 |
| Outcome: Close social relationships (N=1526) |  |  |
| Predictors (genre-specific model) |  |  |
| Multiplayer online battle arena | -0.39 (-0.66 to -0.12) | .02 |
| Shooting | 0.17 (-0.12 to 0.46) | .44 |
| Casual | -0.40 (-0.85 to 0.05) | .18 |
| Action and adventure | -0.56 (-0.91 to -0.22) | .007 |
| Sandbox and simulation | -0.88 (-1.58 to -0.19) | .04 |
| Sports | -0.23 (-0.88 to 0.42) | .62 |
| Strategy | -0.24 (-0.94 to 0.45) | .62 |
| Role-playing | 0.29 (-0.35 to 0.94) | .54 |
| Predictor (overall addiction model) |  |  |
| Overall addiction | -0.34 (-0.54 to -0.14) | .007 |

^a^Game genres denote addiction to corresponding genres.

^b^All models included sex, family economic status, and social media time as covariates; genre-specific models additionally controlled for addiction-to-other-games.

^c^*q* indicates that raw *p* values were adjusted using the Benjamini–Hochberg false discovery rate procedure.
