# Supplementary material for: Genre-Specific Gaming Addiction and Flourishing in Adolescents: Cross-Sectional Survey Study
Source: J Med Internet Res. 2026 Feb 12;28:e89319. doi: 10.2196/89319 (PMC12946777; doi:10.2196/89319)
Supplement: Multimedia Appendix 4 [file jmir_v28i1e89319_app4.docx]

**Table S2.** *E* values for the effects of significant predictors in robust linear regression analyses based on multiple imputed data.

| Variables^a^ | *E* value mean (SD)^b^ | Upper limit mean (SD) |
| --- | --- | --- |
| Outcome: Overall flourishing |  |  |
| Multiplayer online battle arena | 1.31 (0.02) | 1.14 (0.04) |
| Action and adventure | 1.46 (0.01) | 1.33 (0.01) |
| Sandbox and simulation | 1.32 (0.01) | 1.16 (0.02) |
| Overall addiction | 1.48 (0.01) | 1.35 (0.02) |
| Outcome: Happiness and life satisfaction |  |  |
| Action and adventure | 1.34 (0.02) | 1.19 (0.02) |
| Overall addiction | 1.31 (0.01) | 1.15 (0.02) |
| Outcome: Mental and physical health |  |  |
| Action and adventure | 1.42 (0.01) | 1.29 (0.01) |
| Sandbox and simulation | 1.40 (0.01) | 1.27 (0.01) |
| Overall addiction | 1.43 (0.01) | 1.30 (0.01) |
| Outcome: Meaning and purpose |  |  |
| Multiplayer online battle arena | 1.33 (0.02) | 1.17 (0.03) |
| Action and adventure | 1.39 (0.01) | 1.25 (0.02) |
| Overall addiction | 1.48 (0.01) | 1.36 (0.02) |
| Outcome: Character and virtue |  |  |
| Multiplayer online battle arena | 1.32 (0.02) | 1.15 (0.04) |
| Action and adventure | 1.45 (0.02) | 1.32 (0.02) |
| Overall addiction | 1.55 (0.02) | 1.43 (0.02) |
| Outcome: Close social relationships |  |  |
| Action and adventure | 1.40 (0.02) | 1.27 (0.02) |
| Sandbox and simulation | 1.32 (0.03) | 1.17 (0.04) |
| Overall addiction | 1.38 (0.02) | 1.24 (0.02) |

^a^Game genres denote addiction to corresponding genres.

^b^*E* values and their standard deviations are reported on the risk ratio scale; each e-value and its upper limit represent the mean (and standard deviation) across 20 imputed datasets.
